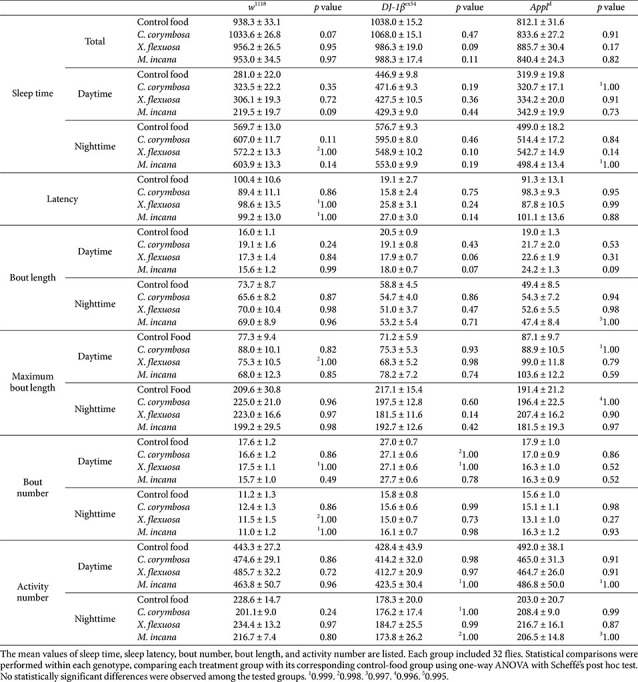# Natural Plant Extracts Rescue Memory Deficits in *Drosophila* Neurodegeneration Models

**DOI:** 10.4014/jmb.2603.03035

**Published:** 2026-06-09

**Authors:** Jiun Sang, Yueyang Kang, Youngseok Lee

**Affiliations:** Department of Integrative Biotechnology, Kookmin University, Seoul 02707, Republic of Korea

**Keywords:** *Drosophila melanogaster*, Memory power, *DJ-1β*, *Appl*, *Casearia corymbosa*, *Xylosma flexuosa*, *Morisonia incana*

## Abstract

Neurodegenerative diseases are characterized by progressive impairments in cognition, stress resilience, and behavior, yet effective therapeutic interventions remain limited. Here, we performed a functional screening of Nicaraguan plant extracts using *Drosophila melanogaster* models of neurodegeneration—specifically *Appl* and *DJ-1β* mutants—to identify candidate extracts with neuroprotective potential. Flies were evaluated using a multi-assay pipeline that included memory testing, reactive oxygen species–induced survival assays, sleep analysis, locomotor performance, and mRNA expression profiling. Screening identified three plant extracts—*Casearia corymbosa*, *Xylosma flexuosa*, and *Morisonia incana*—that significantly improved memory performance in *DJ-1β* mutants, whereas only partial memory recovery was observed in the *Appl* model, indicating genotype-dependent efficacy. None of the extracts rescued sleep defects in either disease model, suggesting limited influence on sleep-regulatory pathways. Notably, all three extracts significantly enhanced survival under oxidative stress in both *Appl* and *DJ-1β* mutants. In addition, *C. corymbosa* and *M. incana* significantly improved locomotor deficits in *DJ-1β* flies. Together, these findings demonstrate that Nicaraguan plant extracts exert selective and dissociable effects on cognitive function and oxidative stress resistance. In particular, *M. incana* emerges as a promising candidate for enhancing cellular stress resilience in neurodegenerative contexts. This work highlights the utility of *Drosophila*-based functional screening for identifying plant-derived compounds with targeted neuroprotective potential.

## Introduction

Major neurodegenerative disorders, particularly Parkinson’s disease and Alzheimer’s disease, represent a growing global health burden driven by aging populations and the absence of effective disease-modifying therapies. Despite their clinical diversity, these disorders share several common pathological features, including progressive cognitive decline, elevated oxidative stress, mitochondrial dysfunction, and disrupted neuronal homeostasis [1-6].

The fruit fly, *Drosophila melanogaster*, has emerged as a powerful and genetically tractable model for studying neurodegenerative mechanisms and for screening candidate neuroprotective interventions [7]. The conservation of cellular stress-response pathways, together with well-established assays for memory, sleep, and stress resistance, enables quantitative evaluation of genotype-dependent phenotypes *in vivo*. These advantages make *Drosophila* particularly suitable for testing complex biological mixtures, such as plant extracts, that are difficult to systematically evaluate in mammalian systems [8-10].

Among available genetic models, mutations in *DJ-1β* and *Appl* provide mechanistically distinct platforms that recapitulate key aspects of Parkinson’s- and Alzheimer’s-related pathology, respectively. Loss of *DJ-1β* disrupts oxidative stress defense and neuronal homeostasis, producing stress sensitivity, dopaminergic neuron degeneration, and progressive behavioral deficits [11-16]. In contrast, *Appl*, the fly ortholog of the human APP gene, plays essential roles in neuronal maintenance and synaptic organization, and its disruption leads to learning and memory impairments reminiscent of Alzheimer’s disease–related dysfunction [17-23].

Plant-derived compounds are increasingly recognized as potential modulators of neurodegenerative processes due to their antioxidant properties and ability to influence cellular stress-response pathways. In this study, we investigated a set of Nicaraguan plant extracts with largely unexplored neurobiological potential. Using *Drosophila* neurodegeneration models, we identified three extracts—*Casearia corymbosa*, *Xylosma flexuosa*, and *Morisonia incana*—that significantly improved memory performance in *DJ-1β* mutants and produced modest improvement in *Appl* mutants. All three extracts also enhanced survival under oxidative stress in both models, whereas none restored sleep defects. These findings suggest selective neuroprotective effects of specific plant extracts and highlight the utility of *Drosophila*-based screening for identifying candidate natural compounds that enhance neuronal stress resilience.

## Materials and Methods

### Fly Stocks

*w^1118^*(BDSC 5905) was used as a control. *Appl^d^* (BDSC 43632) was obtained from the Bloomington *Drosophila* stock center. The *DJ-1β^ex54^* fly line was generously provided by Dr. J. Chung [12].

### Chemical Reagents and Nicaraguan Plant Extracts

Sucrose (CAS No. 57-50-1) and caffeine (CAS No. 58-08-2) were purchased from Sigma-Aldrich (USA). Nicaraguan plant extracts of *Casearia corymbosa* (FBM255-073), *Xylosma flexuosa* (FBM255-074), *Morisonia incana* (FBM255-077), *Zanthoxylum caribaeum* (FBM255-071), *Quercus segoviensis* (FBM255-072), *Cordia collococca* (FBM255-075), *Acanthocereus tetragonus* (FBM255-076), *Casimiroa sapota* (FBM255-078), *Celtis iguanaea* (FBM255-079), *Astronium graveolens* (FBM255-080), *Verbesina pallens* (FBM255-081), *Piscidia grandifolia* (FBM255-082), *Polymnia maculata* (FBM255-083), *Croton cortesianus* (FBM255-084), and *Ardisia revoluta* (FBM255-085) were purchased from the International Biological Material Research Center (Republic of Korea). The Nicaraguan plant extracts were extracted using 99.99% methanol (HPLC grade).

### Taste-Associative Memory Assay

The taste-associative memory assays were performed as described in a previous study [24]. Seven- to eight-day-old male flies were allowed to feed on cornmeal with or without 0.1% Nicaraguan plant extracts. The 0.1% extract concentration was selected as an experimentally optimal condition that enabled stable feeding and sufficient reproducibility across more than four independent behavioral trials for each extract. After 7 days of feeding, the flies were subjected to a 12–18 h fasting period. The flies were then anesthetized with ice, after which they were affixed to glass slides using nail polish. Approximately 10 to 15 flies were employed in each assay. Next, the flies were allowed to recover in a 60% humidified incubator at 25°C for a minimum of 2 h. Our experiment was structured into three distinct phases. The initial phase consisted of a pretest in which a 500 mM sucrose solution was applied to the flies’ legs. Flies exhibiting a positive proboscis extension in response to this stimulus were selected for further testing. This was followed by the training phase, during which 500 mM sucrose was once again applied to the flies’ legs along with the simultaneous exposure to an aversive taste (50 mM caffeine) applied to the labellum as a punishment. Each fly underwent this training process 15 times, and data from the training phase were categorized into three sets of five trials. Upon completion of the training phase, flies were presented with 500 mM sucrose on their legs at multiple points (0, 5, 15, 30, 45, and 60 min). Proboscis extension responses were recorded to assess whether the flies retained the aversive memory associated with the punishment. Memory tests were conducted on control, *DJ-1β^ex54^* and *Appl^d^* flies after being fed either normal food or food supplemented with 0.1% of each Nicaraguan plant extract for 7 days.

### Survival Assay under ROS Stress

Survival assays were performed as described previously [25] with slight modifications. Seven- to eight-day-old male and female flies (15 males and 15 females) were maintained on standard cornmeal food supplemented with or without 0.1% Nicaraguan plant extracts. After 7 days of feeding, the flies were transferred into vials containing 5% sucrose and 1% agar with 1% H_2_O_2_ at 25°C. The dead flies were counted at 1-day intervals, after which they were transferred to a new vial.

### Climbing Assay

Climbing assays were conducted as described previously [26], with some modifications. To evaluate locomotor activity, groups of seven- to eight-day-old 5 male and 5 female flies were collected and anesthetized using CO_2_. The flies were allowed to feed on cornmeal with or without 0.1% Nicaraguan plant extracts. After 7 days of feeding, the flies were then transferred into a graduated cylindrical glass vial (marked at 17.5 cm from the base) and secured with a cotton plug. To initiate the climbing response, the vial was gently tapped 3–4 times against a padded surface, ensuring all flies reached the bottom. Their movement was counted for 1 min. The number of flies that crossed the 17.5 cm threshold was quantified at 10 sec intervals.

### RT-PCR Analysis

Gene expression levels were quantified using reverse transcription quantitative PCR. Thirty 7–8-day-old male flies were allowed to feed on cornmeal with or without 0.1% Nicaraguan plant extracts. After 7 days of feeding, total RNA was extracted from their entire bodies using TRIzol reagent (Invitrogen, USA), followed by cDNA synthesis using AMV transcriptase (Promega, USA). For RT-PCR, specific primer pairs were employed for the *dlp* gene (forward primer: 5’-CAC ATC CCC AGT GGA ATC AC-3’; reverse primer: 5’-TGC CAA CAT TGA TCT GCT TC-3’), with *actin* (forward primer: 5’-CAC CGG TAT CGT TCT GGA CT-3’; reverse primer: 5’-GCG GTG GTG GTG AAA GAG TA-3’) serving as the reference gene. The expression of each gene was quantified using ImageJ (ver. 1.53t; National Institutes of Health, USA). The region of interest containing the DNA was selected and converted to a grey-scale image, after which the image was quantified with ImageJ.

### Sleep Assay

The sleep assays were performed as described previously [27] with slight modifications. *Drosophila* activity monitor systems (DAMs) [28] were employed to automatically track fly activity in 1-min intervals. Activity was logged each time a fly crossed an infrared beam positioned in the middle of the recording tube, which the flies are incapable of perceiving. Flies exhibiting immobility for more than 5 min were classified as being in a state of sleep. The experimental setup included a 12-h light/12-h dark cycle at 25°C, with the aforementioned photoperiod serving as the basis for distinguishing between daytime and nighttime. Seven- to eight-day-old female flies were transferred to vessels containing cornmeal with or without 0.1% Nicaraguan plant extracts. After 7 days of feeding, the flies were transferred into glass tubes containing 1% sucrose in 1% agarose at one end and capped with a cotton ball on the other end. DAMs, equipped with glass tubes, were placed in the incubator 18 h before initiating the recording experiment. Data were collected and averaged over a 2-day period. Latency, defined as the time required to fall asleep after the lights were turned off, was then measured. Bout length was defined as the duration of uninterrupted sleep time, whereas bout number was defined as the number of uninterrupted sleep episodes. Sleep time, bout length, maximum bout length, bout number, and activity number were individually calculated for both daytime and nighttime. 32 flies were analyzed for each experiment.

### Statistical Analysis

The error bars on the graph indicate the standard error of the means (SEMs); pairwise comparisons were conducted using Student’s *t*-test. All statistical analyses were performed using Origin Pro 8 for Windows (ver. 8.0932; Origin Lab Corporation, USA). Single-factor ANOVA coupled with Scheffé’s post hoc test was conducted to compare multiple datasets. Statistical significance was denoted using asterisks (*p < 0.05, **p < 0.01). Survival curves were examined using Kaplan–Meier analysis. Log-rank values were calculated by comparing the outcomes of each experiment.

## Results and Discussion

### Dietary Screening of Nicaraguan Plant Extracts Reveals Candidates that Rescue Taste-Associative Memory Deficits in *DJ-1β* mutant Flies

Parkinson’s disease (PD) is a neurodegenerative disorder classically characterized by progressive motor dysfunction, with the loss or degeneration of dopaminergic neurons (DA) in the substantia nigra representing a defining pathological hallmark [29]. In addition to motor symptoms, PD patients exhibit a broad spectrum of sensory impairments, including deficits in vision [30], nociception [31], olfaction [32], and gustation [24, 33]. In *Drosophila*, dopaminergic neurons play essential roles in diverse behaviors, including locomotion, sleep regulation, feeding, and aversive associative memory. Given the conserved involvement of dopaminergic signaling in reinforcement learning, taste-associative memory paradigms provide a sensitive behavioral readout for detecting early cognitive dysfunction relevant to PD [28, 34, 35].

To identify natural products capable of mitigating taste-associative memory deficits relevant to Parkinson’s disease, we conducted a dietary screening of plant extracts derived from Nicaraguan plants using *DJ-1β* mutant flies to investigate their largely unexplored neurobiological potential (Table 1). Taste-associative memory was assessed using the proboscis extension response (PER) assay, as previously described [24], a well-established Pavlovian conditioning paradigm that provides sensitive detection of impairments in aversive learning and memory in *Drosophila* [36].

**Fig. 1:**
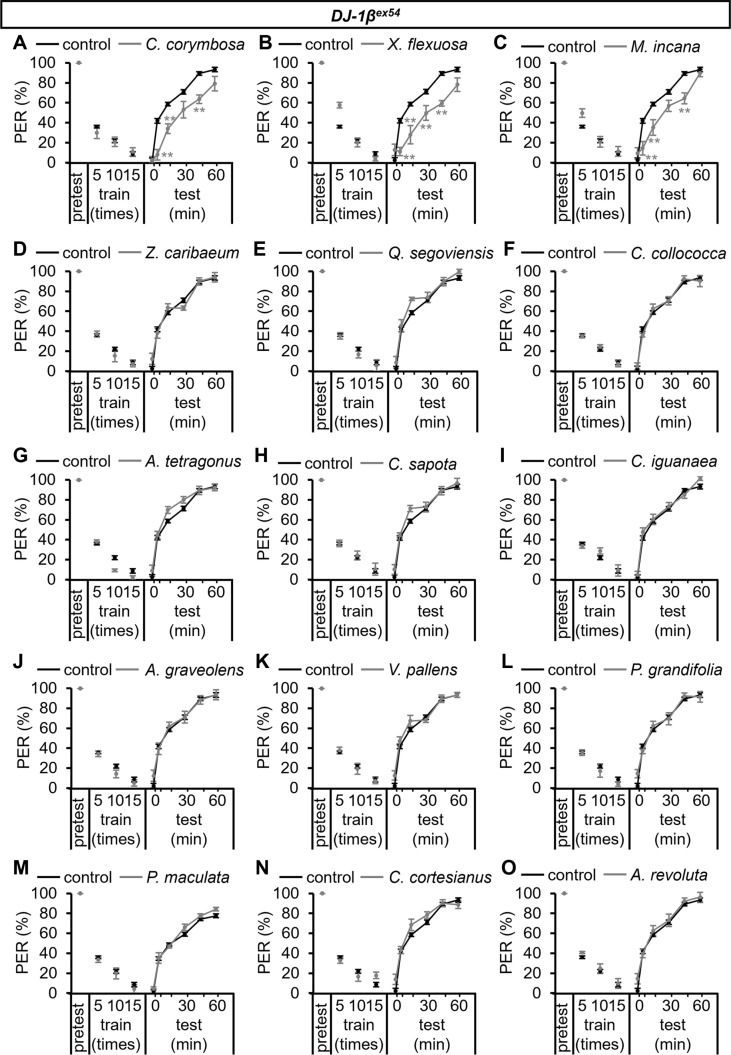
Screening of Nicaraguan plant extracts for their effects on taste-associative memory in *DJ-1β* mutant flies. (**A–O**) Taste-associative memory performance of *DJ-1β* mutants following dietary supplementation with 0.1% Nicaraguan plant extracts: (**A**) *Casearia corymbosa* (*n* = 4), (**B**) *Xylosma flexuosa* (*n* = 4), (**C**) *Morisonia incana* (*n* = 4), (**D**) *Zanthoxylum caribaeum* (*n* = 4), (**E**) *Quercus segoviensis* (*n* = 4), (**F**) *Cordia collococca* (*n* = 4), (**G**) *Acanthocereus tetragonus* (*n* = 4), (**H**) *Casimiroa sapota* (*n* = 4), (**I**) *Celtis iguanaea* extract (*n* = 4), (**J**) *Astronium graveolens* (*n* = 4), (**K**) *Verbesina pallens* (*n* = 4), (**L**) *Piscidia grandifolia* (*n* = 4), (**M**) *Polymnia maculata* (*n* = 4), (**N**) *Croton cortesianus* (*n* = 4), and (**O**) *Ardisia revoluta* (*n* = 4), respectively. The error bars represent SEMs. Statistical significance was assessed using Student’s *t*-test, with **p < 0.01.

Analysis of taste-associative memory performance revealed a selective improvement in *DJ-1β* mutant flies following dietary administration of specific plant extracts (Fig. 1). Dietary administration of *Casearia corymbosa* (Fig. 1A), *Xylosma flexuosa* (Fig. 1B), and *Morisonia incana* (Fig. 1C) resulted in a pronounced and reproducible reduction in PER during the test phase, indicating a significant improvement in aversive taste-associative memory in *DJ-1β* mutant flies. In contrast, the majority of the remaining extracts failed to significantly alter PER responses relative to control-fed *DJ-1β* mutants (Fig. 1D–1O), indicating that memory impairment was not broadly rescued by dietary supplementation and underscoring the stringency of the assay.

Importantly, pretest and training responses remained comparable across groups, suggesting that the reduced PER reflected genuine improvement in associative memory rather than altered sucrose sensitivity or motivation. Together, these results identify *C. corymbosa*, *X. flexuosa*, and *M. incana* as candidate plant extracts capable of ameliorating taste-associative memory deficits in *DJ-1β* mutant flies.

### Candidate Plant Extracts Selectively Improve Taste-Associative Memory in *Appl* Mutant but Not Wild-Type Flies

Cognitive impairment, including deficits in associative learning and memory, represents a shared and early feature of multiple neurodegenerative disease models [1]. In *Drosophila*, both *DJ-1β* and *Appl* mutations have been shown to disrupt neural processes underlying memory formation and retention, through partially distinct molecular mechanisms. Whereas *DJ-1β* dysfunction is closely linked to oxidative stress regulation and dopaminergic signaling, *Appl* mutants primarily model synaptic and neuronal dysfunction associated with amyloid precursor protein processing. Examining whether candidate compounds identified in the *DJ-1β* background exert similar effects in *Appl* mutant flies therefore provides an opportunity to assess the generalizability and disease-context dependence of their memory-modulating properties.

**Fig. 2:**
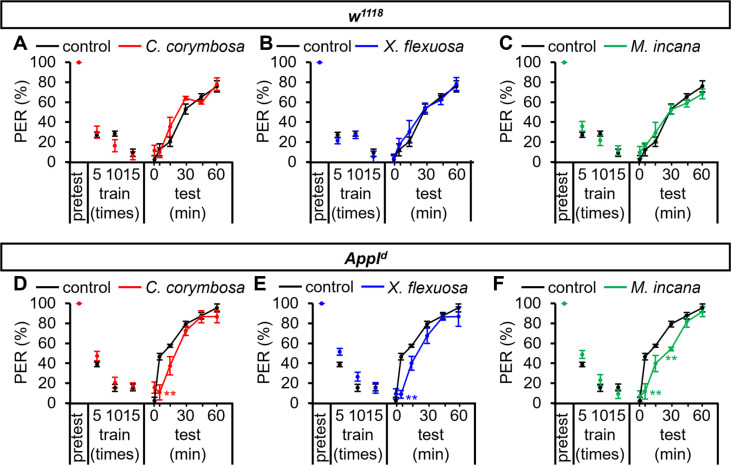
Differential effects of candidate Nicaraguan plant extracts on taste-associative memory in wild-type and *Appl* mutant flies. (**A–C**) Memory performance of *w^1118^* flies following dietary supplementation with 0.1% Nicaraguan plant extracts: (**A**) *Casearia corymbosa* (*n* = 4), (**B**) *Xylosma flexuosa* (*n* = 4), and (**C**) *Morisonia incana* (*n* = 4). (D–F) Memory performance of *Appl* mutant flies under the same conditions: (**D**) *C. corymbosa* (*n* = 4), (**E**) *X. flexuosa* (*n* = 4), and (**F**) *M. incana* (*n* = 4), respectively. The error bars indicate SEMs. Statistical significance was determined using Student’s *t*-test, with **p < 0.01.

To determine whether the memory-restorative effects of the identified candidate extracts extend beyond the *DJ-1β* mutant background, we examined their impact on taste-associative memory in both wild-type and *Appl* mutant flies (Fig. 2). Using the same dietary administration protocol and proboscis extension response (PER) assay described above, we assessed the effects of *C. corymbosa*, *X. flexuosa*, and *M. incana* across these distinct genetic contexts.

In wild-type flies, dietary administration of *C. corymbosa*, *X. flexuosa*, or *M. incana* did not result in a detectable improvement in taste-associative memory during the test phase (Fig. 2A–2C). PER rates during both training and post-training intervals were indistinguishable from those of control-fed flies, indicating that these extracts do not enhance associative memory under physiological baseline conditions.

In contrast, *Appl* mutant flies exhibited a moderate yet reproducible improvement in taste-associative memory following dietary administration of the candidate extracts (Fig. 2D–2F). PER responses were selectively reduced at specific post-training intervals, consistent with partial restoration of aversive memory retention. However, unlike the robust rescue observed in *DJ-1β* mutants (Fig. 1A–1C), the effects in *Appl* mutants were weaker and temporally restricted, suggesting incomplete recovery of cognitive function.

The differential efficacy observed between *DJ-1β* and *Appl* mutant backgrounds may reflect differences in the hierarchical level and nature of the underlying pathology. *DJ-1β* loss directly disrupts core cellular homeostasis, particularly redox balance and mitochondrial function, resulting in elevated oxidative stress and increased cellular vulnerability. In this context, compounds that enhance oxidative stress resistance or stabilize mitochondrial function are expected to exert broad protective effects, consistent with the robust rescue observed in *DJ-1β* mutants.

In contrast, *Appl*-associated phenotypes are primarily linked to defects in synaptic organization, vesicle trafficking, and APP-like protein processing. These processes operate at the level of neuronal connectivity and signaling rather than core metabolic stability and are therefore less directly coupled to acute oxidative stress. As a result, interventions that primarily enhance general cellular resilience may only partially ameliorate *Appl*-related dysfunction, leading to the more modest and temporally restricted effects observed in this study.

Together, these findings support a model in which the candidate plant extracts preferentially target conserved stress-response pathways that are more centrally involved in *DJ-1β*-mediated pathology than in APPL-dependent synaptic dysfunction.

To exclude the possibility that the altered PER responses resulted from nonspecific effects on sensory perception or feeding state, we performed electrophysiological tip recordings from gustatory sensilla on the labellum, the principal peripheral taste organ in *Drosophila* [37]. Recordings were obtained from the sugar-responsive L6 sensillum, which robustly detects sucrose [38], and the bitter-responsive S6 sensillum, which strongly responds to caffeine [39]. Seven-day dietary supplementation with *C. corymbosa*, *X. flexuosa*, or *M. incana* did not significantly alter responses to 500 mM sucrose or 10 mM caffeine in wild-type, *DJ-1β*, or *Appl* mutant flies (Fig. S1A), indicating that peripheral gustatory sensitivity remained intact.

We additionally assessed feeding motivation during the pre-test phase of the PER memory assay. Because flies reliably extend the proboscis to 500 mM sucrose only under a motivated feeding state, comparable pre-test PER responses confirmed that dietary treatment did not alter hunger-driven responsiveness. To further evaluate whether the behavioral phenotypes were secondary to changes in systemic metabolic status, we quantified major energy-related metabolites, including glucose and trehalose as circulating sugars, glycogen as a carbohydrate reserve, and triacylglycerol (TAG) as a lipid storage marker. Dietary supplementation with the candidate extracts did not induce consistent changes in these metabolic parameters across wild-type, *DJ-1β*, or *Appl* mutant flies (Fig. S1B–S1D).

Together, these control experiments demonstrate that the reduced PER responses observed during the test phase are unlikely to arise from altered taste sensitivity, feeding motivation, or systemic metabolic changes. Instead, the data support the conclusion that the candidate extracts specifically improve neural processes underlying taste-associative memory in neurodegenerative disease models.

Furthermore, an important limitation of the present study is that all candidate plants were evaluated as crude methanolic extracts without direct chemical fractionation or compound-level validation. Accordingly, the current findings should be interpreted as functional evidence at the extract level rather than definitive mechanistic evidence attributable to specific molecules. Nevertheless, previous phytochemical studies provide a preliminary framework for understanding the potential bioactive basis of the observed neuroprotective effects (Table 2). Bioassay-guided fractionation of *C. corymbosa* has identified several diterpene- and phenolic-related constituents, including casearborin C, syringic acid, *ent*-3β-hydroxy-(-)-13-*epi*-manoyl oxide, *ent*-(-)-13-*epi*-manoyl oxide, *ent*-(-)-kaur-16-en-19-oic acid, and γ-sitosterol [40]. Likewise, *X. flexuosa* has been reported to contain xylosmin, 2′-benzoylpoliothrysoside, salireposide, and lupeol [41], compounds previously associated with antioxidant or anti-inflammatory activities. In contrast, the phytochemical composition of *M. incana* remains largely unresolved. To infer potential bioactive properties, we therefore considered related species within the closely associated *Morisonia*/*Capparis* taxonomic group [42]. For example, *Morisonia peruviana* contains 11-methylsulfonylundecyl glucosinolate, a glucosinolate derivative reported to suppress tumor growth [43], while several *Capparis* species exhibit antioxidant, anti-inflammatory, and cytoprotective activities [44]. Notably, extracts from *C. spinosa* selectively induce ROS-mediated apoptosis in cancer cells while sparing normal tissues, and glucosinolate-derived metabolites have been broadly implicated in stress-responsive and protective cellular pathways [45]. Although these reports do not directly establish the mechanisms underlying the memory-restorative effects observed here, they collectively suggest that the candidate extracts contain chemically diverse metabolites with plausible neuroprotective potential. Although the phytochemical composition of each extract is distinct, several shared chemical features may account for the consistently observed enhancement of oxidative stress resistance. Notably, both *C. corymbosa* and *X. flexuosa* contain phenolic compounds and terpenoid derivatives, including diterpenes and triterpenes, which have been widely associated with antioxidant activity and modulation of cellular redox homeostasis. In addition, sterol-related compounds such as γ-sitosterol and triterpenoids like lupeol have been reported to exert cytoprotective and anti-inflammatory effects in various systems. While the chemical composition of *M. incana* remains less well defined, related species within the *Morisonia*/*Capparis* group are enriched in glucosinolate derivatives and other stress-responsive metabolites that can influence redox signaling pathways.

Despite their structural diversity, these compounds may converge functionally on common mechanisms such as reactive oxygen species buffering, mitochondrial stabilization, or activation of endogenous stress-response pathways. This convergence at the level of cellular stress regulation may underlie the shared ability of the three extracts to enhance oxidative stress resistance, while differences in compound composition could contribute to the observed variation in behavioral efficacy across genotypes. Future studies combining bioactivity-guided fractionation, metabolomic profiling, and compound-specific validation will therefore be necessary to identify the active constituents and determine whether the differential efficacy observed between *DJ-1β* and *Appl* mutant backgrounds reflects selective modulation of distinct neurodegenerative pathways.

### Candidate Plant Extracts Differentially Modulate Oxidative Stress Resistance in *DJ-1β* Mutant and *Appl* Mutant Flies

*DJ-1β* contributes to neuronal resistance against oxidative stress–induced apoptosis by regulating Daxx-like protein (DLP) expression and subcellular distribution, linking *DJ-1β* dysfunction to dopaminergic neuron loss in Parkinson’s disease. In *Drosophila*, DLP acts as a pro-apoptotic regulator and JNK pathway activator, analogous to mammalian Daxx, whose expression is induced by oxidative stress and UV exposure. Consistently, H_2_O_2_ and UV treatment elevate DLP expression, reinforcing its role in stress-activated apoptotic signaling [16]. Accordingly, we next assessed whether the candidate plant extracts identified in behavioral assays influence oxidative stress resistance at both physiological and molecular levels.

**Fig. 3:**
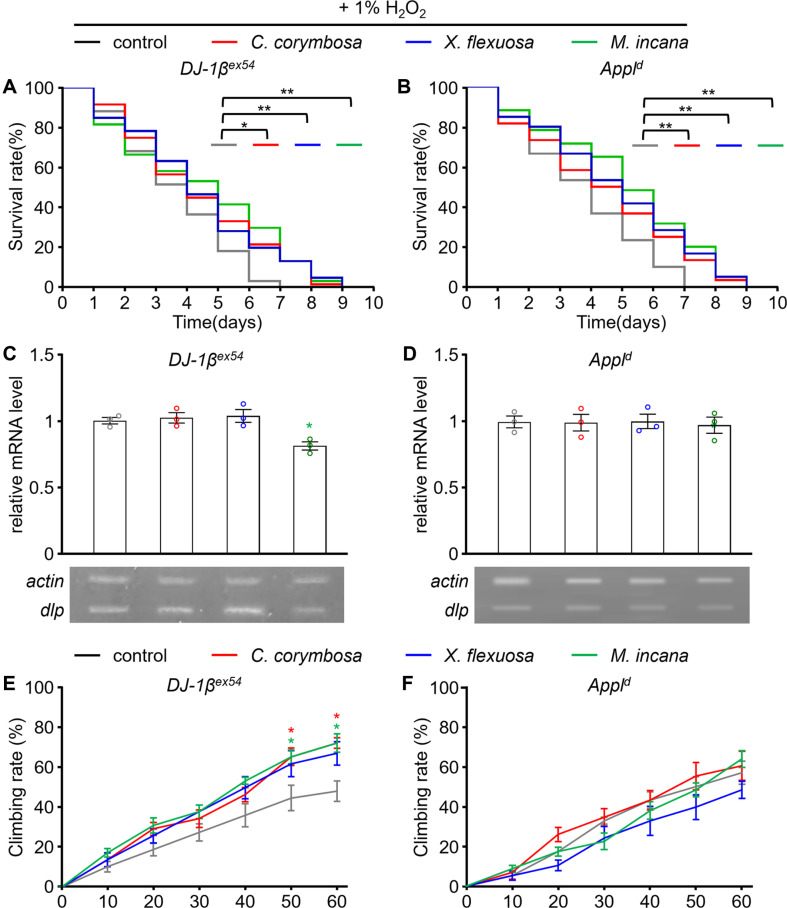
Candidate plant extracts differentially modulate oxidative stress resistance, stress-related gene expression, and locomotor performance in *DJ-1β* and *Appl* mutant flies. (**A–B**) Effects of dietary supplementation with 0.1% *Casearia corymbosa*, *Xylosma flexuosa*, or *Morisonia incana* on survival under oxidative stress (1% H_2_O_2_) in (**A**) *DJ-1β* and (**B**) *Appl* mutants (3 trials; 20 flies per trial). (**C–D**) Relative expression levels of *dlp* and *actin* in (**C**) *DJ-1β* and (**D**) *Appl* mutants following extract supplementation (*n* = 4). (**E–F**) Climbing performance of (**E**) *DJ-1β* and (**F**) *Appl* mutants under the same conditions (6 trials; 10 flies per trial). Error bars represent SEMs and each dot indicates the distribution of individual sample values. (**A and B**) Survival curves were analyzed using the Kaplan–Meier product-limit method, and differences between groups were evaluated using the log-rank (Mantel–Cox) tests. (**C–F**) Multiple experimental groups were compared via single-factor ANOVA and Scheffé’s *post hoc* test. Statistical significance is denoted by asterisks (*p < 0.05, **p < 0.01).

Survival analyses under oxidative stress conditions revealed that dietary administration of *C. corymbosa*, *X. flexuosa*, and *M. incana* markedly extended lifespan in *DJ-1β* mutant flies (Fig. 3A). A similar overall pattern was observed in *Appl* mutant flies, in which each plant extract again produced the most pronounced extension of survival under oxidative stress (Fig. 3B). Together, these findings suggest that *C. corymbosa*, *X. flexuosa*, and *M. incana* enhance cellular resilience to oxidative stress in neurodegenerative disease models, potentially through modulation of conserved stress-response pathways.

To determine whether the observed survival benefits were accompanied by transcriptional changes in stress-related genes, we quantified relative *dlp* mRNA expression following dietary administration of the candidate extracts (Fig. 3C and 3D). Although all tested extracts significantly extended survival under oxidative stress, only *M. incana* treatment produced a corresponding reduction in *dlp* mRNA levels in *DJ-1β* mutant flies (Fig. 3C), whereas no such effect was observed in *Appl* mutants (Fig. 3D). The divergence between transcriptional output and physiological protection suggests that *dlp* regulation alone cannot fully account for the extract-mediated improvement in oxidative stress resistance. This dissociation further indicates that the protective effects of the candidate extracts are at least partly mediated through *dlp*-independent pathways, potentially involving parallel stress-response or mitochondrial regulatory mechanisms. Because the RT-PCR analysis was performed using whole-fly RNA, the observed reduction in *dlp* expression reflects a global organismal response rather than a tissue- or neuron-specific change. Nevertheless, the detectable decrease in whole-fly *dlp* transcript levels indicates that *M. incana* feeding is associated with a measurable shift in stress-related molecular status in *DJ-1β* mutants under these experimental conditions. Notably, while the improved oxidative stress resistance observed in *DJ-1β* mutants may be tentatively associated with reduced *dlp* expression, this relationship remains correlative and should be interpreted with caution given the use of whole-fly RT-PCR without tissue-specific resolution. In contrast, the enhanced survival observed in *Appl* mutants under oxidative stress appears to arise through *dlp*-independent mechanisms.

Since progressive loss of motor coordination is a hallmark of neurodegenerative pathology, we further investigated whether the candidate extracts could mitigate locomotor deficits using a climbing assay [36, 23]. In *DJ-1β* mutants, dietary supplementation with *C. corymbosa* or *M. incana* significantly rescued the impaired climbing ability compared to the control-fed group (Fig. 3E), whereas in *Appl* mutants, locomotor improvements were not observed with any of the three candidate extracts (Fig. 3F), indicating that the extracts do not restore circuit-level motor function in this genetic context despite improving cellular stress resistance.

This dissociation between improved oxidative stress resistance and the absence of locomotor rescue in *Appl* mutants suggests that enhanced cellular stress tolerance alone is insufficient to restore circuit-level neuronal function in this context. In contrast, the broader phenotypic rescue observed in *DJ-1β* mutants is consistent with the idea that targeting core cellular stress pathways can produce system-wide functional improvements when pathology is driven by intrinsic cellular vulnerability.

Despite the reproducible improvement in memory, locomotor behavior, and oxidative stress resistance following dietary supplementation with the candidate extracts, the precise molecular mechanisms underlying these protective effects remain incompletely defined. In the present study, *dlp* expression was examined as an initial molecular readout associated with stress-related pathology, particularly in the *DJ-1β* background. However, the divergence between transcriptional changes and physiological rescue indicates that *dlp* regulation alone cannot fully explain the observed phenotypic improvements.

The extracts also improved multiple disease-associated phenotypes, including associative memory, oxidative stress resistance, and locomotor activity, suggesting a broader influence on neuronal resilience or cellular stress adaptation.

*C. corymbosa* and *X. flexuosa* improved several disease-associated phenotypes, including associative memory, oxidative stress survival, and locomotor performance, in both *DJ-1β* and *Appl* mutant backgrounds. These findings suggest that the extracts may partially alleviate neurodegenerative dysfunction rather than simply producing nonspecific physiological effects. However, unlike *M. incana*, neither *C. corymbosa* nor *X. flexuosa* significantly altered *dlp* expression under our experimental conditions. This discrepancy indicates that their protective effects are unlikely to be mediated primarily through the *dlp*-related pathway and instead may involve alternative molecular targets or parallel stress-response mechanisms. Because both *DJ-1β* and *Appl* mutants exhibit complex and multifactorial pathological features, it is plausible that these extracts influence broader pathways associated with neuronal resilience, oxidative stress adaptation, or synaptic function.

Overall, the present study establishes a functional framework for the neuroprotective effects of these Nicaraguan plant extracts, while highlighting the need for mechanistic dissection of the underlying pathways. To directly test the proposed model, future studies should incorporate genetic epistasis approaches targeting key oxidative stress regulators, including CncC/Nrf2, JNK signaling components, and DLP. In parallel, tissue-specific manipulations using neuron- or gut-restricted drivers will be important for resolving the relative contribution of central versus peripheral mechanisms. Complementary analyses of mitochondrial function, reactive oxygen species levels, and synaptic integrity will further clarify whether these extracts preferentially act on cellular metabolic resilience or higher-order neuronal processes.

### Sleep Behavior in Wild-Type and Neurodegenerative Models

Sleep disturbances are commonly reported in individuals with PD and include disrupted sleep architecture, altered sleep–wake rhythms, insomnia, sleep fragmentation, and reduced sleep quality [46]. Although they lack eyelids, *Drosophila* exhibit robust circadian rhythmicity and well-defined sleep–wake cycles, supporting their use as a model for studying sleep regulation in neurodegenerative disease contexts. Given the close relationship between sleep regulation, dopaminergic signaling, and cognitive function, it is important to determine whether dietary compounds that influence memory performance or stress resistance also alter sleep architecture. In *Drosophila*, sleep-like states are defined by prolonged immobility, circadian regulation, and homeostatic control, making flies a powerful model for investigating the genetic and molecular basis of sleep regulation [47].

**Fig. 4:**
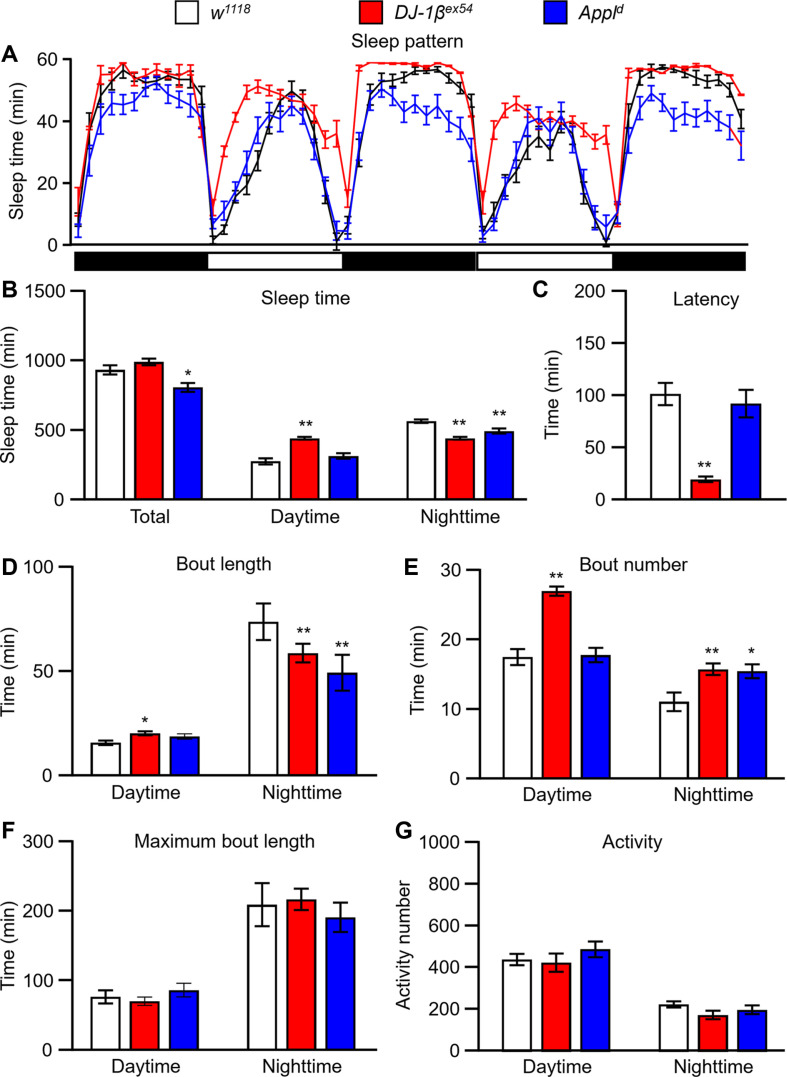
Sleep phenotypes of wild-type (*w^1118^*), *DJ-1β^ex54^*, and *Appl^d^* flies under control diet conditions. Seven- to eight-day-old female flies were maintained on a control cornmeal diet. Sleep was assessed using 1% sucrose and 1% agarose medium (n = 32 per genotype). (**A**) Sleep profiles of wild-type, *DJ-1β^ex54^*, and *Appl^d^* flies under control conditions; white and black bars indicate daytime and nighttime, respectively. (**B**) Total sleep duration during day and night. (**C**) Nighttime sleep latency. (**D**) Mean daytime and nighttime sleep bout length. (**E**) Sleep bout number during day and night. (**F**) Maximum daytime and nighttime sleep bout length. (**G**) Locomotor activity during day and night. Multiple experimental groups were compared with *w^1118^* via single-factor ANOVA and Scheffé’s *post hoc* test. Statistical significance is denoted by asterisks (*p < 0.05, **p < 0.01).

However, sleep regulation has not previously been examined in *Appl* mutants. Therefore, we analyzed sleep parameters in two mutants (*DJ-1β* and *Appl*) and control flies following dietary administration of *C. corymbosa*, *X. flexuosa*, and *M. incana* extracts (Fig. 4 and Table 3). Sleep was monitored for 2.5 days to calculate the average number of sleep episodes (Fig. 4A). *DJ-1β* mutants displayed increased daytime sleep but reduced nighttime sleep (Fig. 4B). In contrast, *Appl* mutants showed reduced sleep during the nighttime period (Fig. 4B). Additionally, *DJ-1β* mutants exhibited markedly shorter sleep latency (Fig. 4C). Daytime bout length was slightly increased in *DJ-1β* mutants, whereas nighttime bout length was significantly reduced in both mutant strains (Fig. 4D). Interestingly, the number of sleep bouts was significantly increased during the daytime in *DJ-1β* mutants, and during the nighttime in both mutants (Fig. 4E). The maximum bout length and overall locomotor activity in both mutants were comparable to those of control flies (Fig. 4F and 4G).

To evaluate whether the candidate plant extracts exert nonspecific sedative, stimulatory, or circadian-disrupting effects, we performed a comprehensive sleep analysis following dietary treatment with *C. corymbosa*, *X. flexuosa*, and *M. incana*. Sleep parameters were assessed under baseline conditions in wild-type, *DJ-1β*, and *Appl* mutant flies. Quantitative analysis revealed no significant improvement or alteration in sleep episodes in either mutants or controls following extract administration (Table 3).

Together, the consistent absence of sleep-related effects across wild-type and mutant genotypes provides an important control. These findings indicate that the improvements observed in taste-associative memory and oxidative stress resistance are unlikely to arise from indirect changes in sleep regulation.

## Conclusion

Our findings identify *C. corymbosa*, *X. flexuosa*, and *M. incana* as candidate plant extracts capable of ameliorating taste-associative memory deficits in *Drosophila* models of neurodegeneration. The observed efficacy was genotype dependent, with stronger effects in *DJ-1β* mutants than in *Appl* mutants, suggesting that these extracts may preferentially act on pathological pathways more prominently engaged in the *DJ-1β* model. Importantly, none of the candidate extracts produced detectable alterations in sleep architecture or baseline locomotor activity across wild-type or mutant backgrounds, indicating that the improvements in memory performance are unlikely to result from nonspecific behavioral modulation.

Among the candidates, *M. incana* showed the most consistent protective profile. Dietary administration enhanced resistance to oxidative stress, partially improved locomotor performance, and reduced whole fly *dlp* mRNA levels in *DJ-1β* mutant flies, suggesting a potential role in strengthening cellular stress-defense mechanisms. Although the precise molecular pathways remain to be elucidated, these findings highlight the value of *Drosophila*-based behavioral and physiological assays for identifying dietary plant extracts with selective neuroprotective potential. Future studies aimed at defining the underlying molecular targets and evaluating the long-term efficacy of these extracts will be important for assessing their translational relevance in the context of neurodegenerative disorders.

## Supplemental Materials

Supplementary data for this paper are available on-line only at http://jmb.or.kr.



## Figures and Tables

**Table 1:** Examined Nicaraguan plant extracts and their associated functional properties.

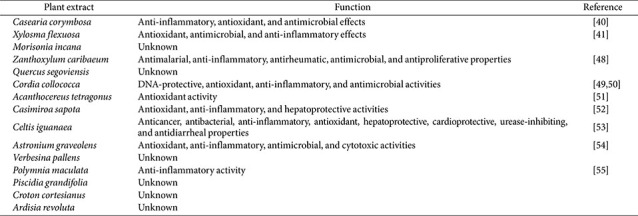

**Table 2:** Reported phytochemical composition and biological activity of candidate plant extracts.

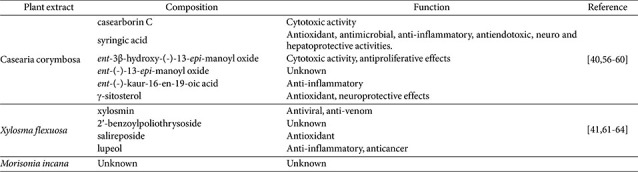

**Table 3:** Quantitative analysis of sleep parameters in wild-type, *Appl*, and *DJ-1β* mutant flies under control diet and plant extract feeding conditions.