# Supplementary material for: Natural Plant Extracts Rescue Memory Deficits in Drosophila Neurodegeneration Models
Source: J Microbiol Biotechnol. 2026 Jun 9;36:e2603035. doi: 10.4014/jmb.2603.03035 (PMC13275258; doi:10.4014/jmb.2603.03035)
Supplement: Supplementary file 1 [file jmb-36-e2603035-supple.pdf]

Methods

1. Tip recording assay

To measure tastant-induced action potentials, we performed tip recordings as previously described [1]. Seven- to eight-day-old male flies were allowed to feed on cornmeal with or without 0.1% Nicaraguan plant extracts. After 7 days of feeding, we immobilized flies by exposing them to ice. We immobilized a fly by inserting a reference glass electrode filled with Ringer's solution through the back thorax all the way into the proboscis. The recording glass electrode (tip diameter 10–20 μm) contained either 500 mM sucrose or 10 mM caffeine as tastant compounds, dissolved in distilled water with 30 mM tricholine citrate (TCC) or 1 mM KCl as the electrolyte, respectively. Reference glass electrode and recording glass electrode were created by processing Standard Glass Capillaries (Cat # IB150F-3, World Precision Instruments, USA) with glass puller. The recording electrode was placed over a bristle on the labellum and connected to a pre-amplifier (Taste PROBE, Syntech, Germany), which amplified the signals by a factor of 10 using a signal connection interface box (Syntech) and a 100–3000 Hz band-pass filter. The recorded action potentials were acquired at a sampling rate of 12 kHz and analyzed using Autospike 3.1 software (Syntech). The average frequencies of action potentials (spikes/s) were based on spikes occurring between 50 ms and 550 ms after contact of the recording electrode. The sensilla bristles were defined as described [2].

2. TAG level measurements

TAG level quantification was performed as described previously [3] using a LiquiColor Triglyceride Test kit (Cat No. 2100-225; Stanbio Laboratory, Germany) with some

modifications. Seven- to eight-day-old male flies were allowed to feed on cornmeal with or without 0.1% Nicaraguan plant extracts. After 7 days of feeding, samples from 10 male flies were weighed and crushed in 1 ml of PBST (1X PBS and 0.2% Triton X-100). The homogenate was centrifuged at ~9,500g for 3 min. Afterward, 100 μl of the supernatant was added to 1 ml of Stanbio LiquiColor Triglyceride Test kit reagent or 1 ml of deionized water, which was used as a baseline. The mixture was incubated at 37°C for 15 min, after which absorbance was recorded at 500 nm and compared with a standard calibration curve.

3. Glycogen measurements

Glycogen levels in whole fly extracts were measured as described in a previous study [4]. Seven- to eight-day-old male flies were allowed to feed on cornmeal with or without 0.1% Nicaraguan plant extracts. After 7 days of feeding, 5 male flies were weighed and homogenized in 100 μl of ice-cold phosphate buffered saline (1X PBS). The enzymes were then inactivated by incubating the homogenates at 70°C for 5 min, after which the samples were centrifuged at 12,500g for 3 min at 4°C. Then, 20 μl of the supernatants were transferred to 1.5 ml tubes and diluted 1:3 in 1X PBS. Afterward, 1.5 μl of amyloglucosidase suspension was diluted in 998.5 μl 1X PBS, and 20 μl of the diluted amyloglucosidase solution was added to 20 μl aliquots of each test sample (and to glycogen standards) to convert the glycogen into glucose. The samples and glycogen standards were incubated at 37°C for 60 min. A commercial glucose (HK) assay reagent (G3293 VER) was then used to measure total glucose at 340 nm. To determine the

glycogen concentrations, the glucose levels in the test samples were compared with a standard curve derived from converting glycogen standards to glucose.

4. Trehalose and glucose measurements in tissue

Quantification of trehalose and glucose levels in whole fly extracts was performed as described previously [5]. Seven- to eight-day-old male flies were allowed to feed on cornmeal with or without 0.1% Nicaraguan plant extracts. After 7 days of feeding, 10 male flies were weighed and homogenized in 250 µl of 0.25 M Na_2CO_3 buffer and incubated in a water bath at 95°C for 5 min to inactivate all enzymes. Next, 150 µl of 1 M acetic acid and 600 µl of 0.25 M sodium acetate (pH 5.2) were added, and the solution was centrifuged (10 min, 12,500g, 24°C). Overnight, 200 µl of each supernatant was incubated at 37°C with 2 µl porcine kidney trehalase (T8778 UN; Sigma-Aldrich) to convert trehalose into glucose. Then, 100 µl of this solution was added to 1 ml of glucose hexokinase solution (GAHK-20; Sigma-Aldrich) and incubated for 20 min at 37°C. Glucose levels were quantified at 340 nm. Glucose concentrations were quantified using a glucose standard curve.

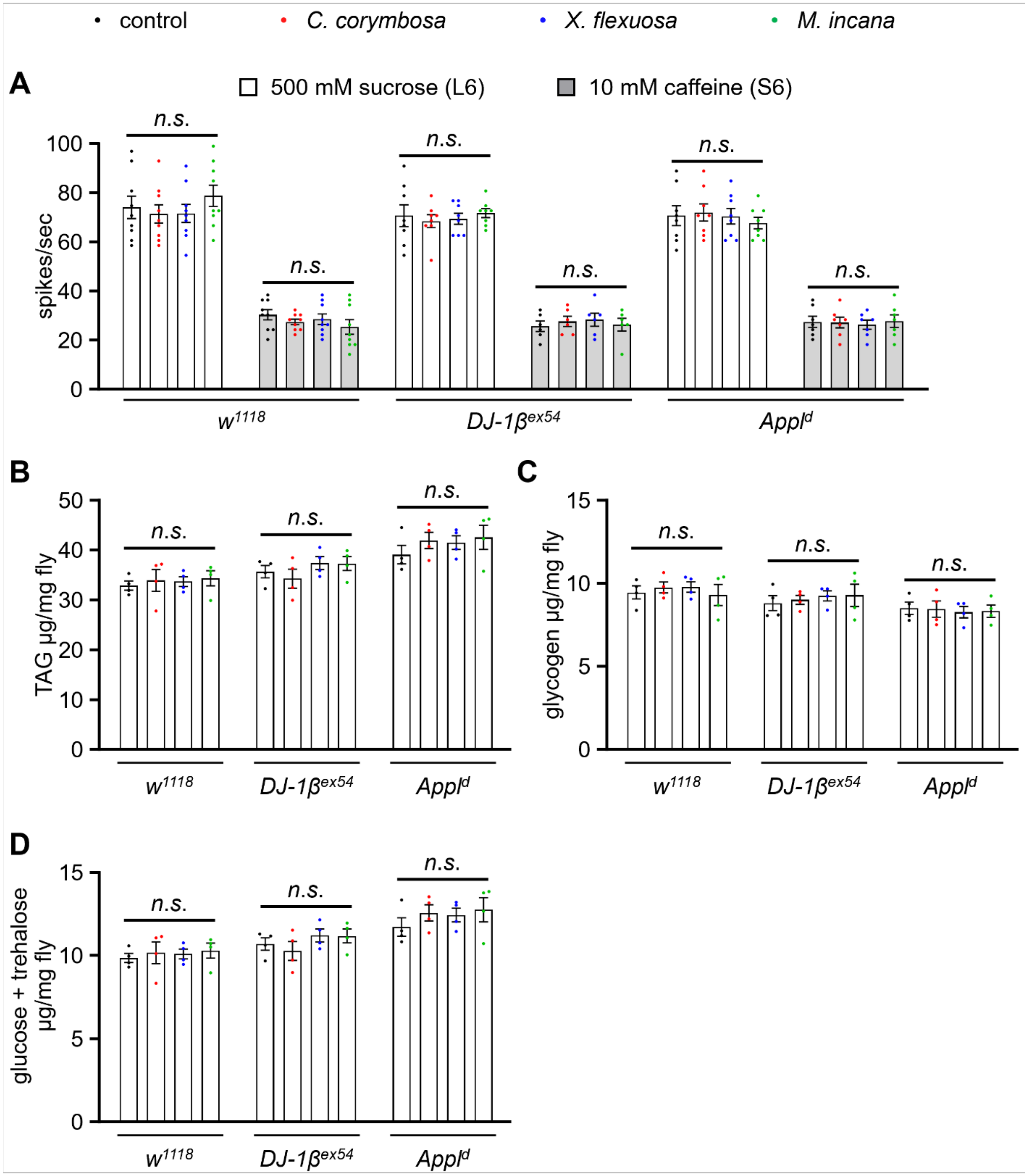
control
C. corymbosa
X. flexuosa
M. incana
A
500 mM sucrose (L6)
10 mM caffeine (S6)
spikes/sec
n.s.
w1118
DJ-1βex54
Appld
B
TAG µg/mg fly
C
glycogen µg/mg fly
D
glucose + trehalose µg/mg fly

Figure legends

Fig. S1. Dietary supplementation with candidate plant extracts does not alter peripheral gustatory sensitivity or systemic metabolic status. Wild-type, *DJ-1β* mutant, and *Appl* mutant flies were maintained on control food or food supplemented with *C. corymbosa*, *X. flexuosa*, or *M. incana*. (A) Peripheral taste responses were assessed by electrophysiological tip recordings from the sugar-responsive L6 sensillum stimulated with 500 mM sucrose and the bitter-responsive S6 sensillum stimulated with 10 mM caffeine. Average spike frequencies (spikes/s) are shown (n = 6–8). (B) Whole-body triacylglycerol (TAG) levels (n = 4). (C) Glycogen levels (n = 4). (D) Glucose and trehalose levels (n = 4). Statistical comparisons among dietary treatment groups within each genotype were performed using one-way ANOVA followed by Scheffé’s post hoc test. No significant differences were detected following extract supplementation, indicating that the candidate extracts do not measurably affect peripheral gustatory responsiveness or basal metabolic parameters under the conditions tested.

References

1. Lee Y, Moon SJ, Montell C. 2009. Multiple gustatory receptors required for the caffeine response in *Drosophila*. Proc. Natl. Acad. Sci. 106: 4495–4500.

2. Weiss LA, Dahanukar A, Kwon JY, Banerjee D, Carlson JR. 2011. The Molecular and Cellular Basis of Bitter Taste in *Drosophila*. Neuron 69: 258–272.

3. De Truchis P, Kirstetter M, Perier A, Meunier C, Zucman D, Force G, et al. 2007. Reduction in Triglyceride Level With N-3 Polyunsaturated Fatty Acids in HIV-Infected Patients Taking Potent Antiretroviral Therapy: A Randomized Prospective Study. JAIDS J. Acquir. Immune Defic. Syndr. 44: 278.

4. Dus M, Min S, Keene AC, Lee GY, Suh GSB. 2011. Taste-independent detection of the caloric content of sugar in *Drosophila*. Proc. Natl. Acad. Sci. 108: 11644–11649.

5. Meunier N, Belgacem YH, Martin J-R. 2007. Regulation of feeding behaviour and locomotor activity by takeout in *Drosophila*. J. Exp. Biol. 210: 1424–1434.
